# Advances and Applications of Carbon Nanotubes

**DOI:** 10.3390/nano13192674

**Published:** 2023-09-29

**Authors:** Simone Morais

**Affiliations:** REQUIMTE/LAQV, ISEP, Polytechnic of Porto, rua Dr. António Bernardino de Almeida, 4249-015 Porto, Portugal; sbm@isep.ipp.pt

Carbon nanotubes (CNT) (single-walled CNT, multiwalled CNT, non-covalently functionalized and covalently functionalized CNT, and/or CNT tailored with chemical or biological recognition elements) are by far the most popular nanomaterials thanks to their high electrical and thermal conductivities and mechanical strength, specific optical and sorption properties, low cost, and easy preparation, among other interesting characteristics [1,2,3,4]. Current applications comprise the use of CNT-based building blocks in the design of (mechanical, thermal, optical, magnetic, chemical, and biological) sensors, pre-concentration and clean-up schemes in analytical chemistry, (electro)catalysis, environmental protection (e.g., water quality control and treatment, adsorption of contaminants, desalinization, etc.), energy conversion including batteries, electromagnetic absorption and shielding materials, and in other novel tailored (nano)composites or hybrid nanostructures. However, other potential applications are constantly being discovered by ongoing investigations. Thus, the main aim of this Topic is to outline the recent advances in and applications of CNT by gathering a set of multidisciplinary research articles.

In total, twenty-three articles are published on this Topic in the participating journals *Nanomaterials*, *Applied Sciences*, and *Materials*. The presented knowledge embraces numerical simulations [2,5,6,7,8,9,10,11] and/or experimental data [1,12,13,14,15,16,17,18,19,20,21,22,23].

The research community has been particularly active in modelling and simulating different scenarios to better understand the behaviour of natural convection, energy absorption, and thermal and mass transport, as well as the mechanical, electronic, and optical properties.

Khan et al. [2,9] conducted a thorough numeral analysis of the heat transfer and fluid movement between plates under the influence of magnetic fields; for this purpose, a hybrid nanofluid composed of CNT, ferrous oxide, and water was used. Alsabery et al. [6] also studied convective flow and heat transfer, but by using alumina nanoparticles water-based nanofluid inside a cavity with vertical walls. These characterizations [2,6,9] are particularly important for the design of insulation systems, solar energy storage, and cooling systems, among others. 

Molecular dynamic simulations have provided deep theoretical insights into the heat transfer mechanisms of branched CNT [5] and improved the tribological features of CNT/vulcanized natural rubber composites for aeronautics [8] and the energy absorption capacity of CNT buckypaper [11]. An additional study [7] used density functional theory to explore the combination of CNT and 2D monolayer germanium selenide (a semiconductor) for potential optoelectronic applications. Numerical and experimental methods were coupled to assess the impact of path planning methods on the mechanical performance of the components [10]. All these theoretical studies pave the way for novel applications of CNT, particularly in the engineering field and in industry.

The other articles on this Topic delve into different experimental approaches for the synthesis of new (nano)materials or the development of new analytical tools. A large set of new (nano)materials is under scrutiny, namely, silica-microsphere-supported N-doped CNT for electromagnetic wave absorption [13], low crystallinity CNT arrays as potential gas sensors [14], synthesis of tin dioxide/CNT nanonests for use in lithium-ion batteries [15], 3D CNT monoliths for controlled adsorption [16], and six different CNT yarns prepared by various spinning methods [17], among others [18,19]. Further developments were focused on materials for civil infrastructures such as sand–gel composites [20], cementitious composites [4,21], and ultra-high-performance concrete [23].

It is worth mentioning that analytical strategies were also addressed in this Topic by designing a novel biosensor based on SWNT/DNAzyme for calcium determination in milk [22] and characterization of the photothermal features of CNT films [3]. 

The remarkable applications of CNT are already uncountable, but their potential is undoubtedly yet to be fully developed. Advances in their synthesis, characterization, and application, as concluded by reading the reported studies on this Topic, involve merging multi- and transdisciplinary knowledge ranging from fundamental science to technological innovations.

## Data Availability

No new data were created in this study. Data sharing is not applicable to this article.
